# Photoreceptor Layer Thickness Changes During Dark Adaptation Observed With Ultrahigh-Resolution Optical Coherence Tomography

**DOI:** 10.1167/iovs.17-22171

**Published:** 2017-09

**Authors:** Chen D. Lu, ByungKun Lee, Julia Schottenhamml, Andreas Maier, Edward N. Pugh, James G. Fujimoto

**Affiliations:** 1 Research Laboratory of Electronics and Department of Electrical Engineering and Computer Science, Massachusetts Institute of Technology, Cambridge, Massachusetts, United States; 2 Pattern Recognition Laboratory, Friedrich-Alexander University Erlangen-Nürnberg, Erlangen, Germany; 3 Departments of Physiology and Membrane Biology, and Cell Biology and Human Anatomy, School of Medicine, University of California-Davis, Davis, California, United States

**Keywords:** optical coherence tomography, dark adaptation, outer segments, photoreceptors

## Abstract

**Purpose:**

To examine outer retinal band changes after flash stimulus and subsequent dark adaptation with ultrahigh-resolution optical coherence tomography (UHR-OCT).

**Methods:**

Five dark-adapted left eyes of five normal subjects were imaged with 3-μm axial-resolution UHR-OCT during 30 minutes of dark adaptation following 96%, 54%, 23%, and 0% full-field and 54% half-field rhodopsin bleach. We identified the ellipsoid zone inner segment/outer segment (EZ[IS/OS]), cone interdigitation zone (CIZ), rod interdigitation zone (RIZ), retinal pigment epithelium (RPE), and Bruch's membrane (BM) axial positions and generated two-dimensional thickness maps of the EZ(IS/OS) to the four bands. The average thickness over an area of the thickness map was compared against that of the dark-adapted baselines. The time-dependent thickness changes (photoresponses) were statistically compared against 0% bleach. Dark adaptometry was performed with the same bleaching protocol.

**Results:**

The EZ(IS/OS)-CIZ photoresponse was significantly different at 96% (*P* < 0.0001) and 54% (*P* = 0.006) bleach. At all three bleaching levels, the EZ(IS/OS)-RIZ, -RPE, and -BM responses were significantly different (*P* < 0.0001). The EZ(IS/OS)-CIZ and EZ(IS/OS)-RIZ time courses were similar to the recovery of rod- and cone-mediated sensitivity, respectively, measured with dark adaptometry. The maximal EZ(IS/OS)-CIZ and EZ(IS/OS)-RIZ response magnitudes doubled from 54% to 96% bleach. Both EZ(IS/OS)-RPE and EZ(IS/OS)-BM responses resembled dampened oscillations that were graded in amplitude and duration with bleaching intensity. Half-field photoresponses were localized to the stimulated retina.

**Conclusions:**

With noninvasive, near-infrared UHR-OCT, we characterized three distinct, spatially localized photoresponses in the outer retinal bands. These photoresponses have potential value as physical correlates of photoreceptor function.

Optical coherence tomography (OCT) is a noninvasive, noncontact method of visualizing the various layers of the retina in depth.^1^ OCT has become a standard tool in ophthalmology for diagnosing and monitoring the treatment of retinal diseases. With the advent of ultrahigh-resolution OCT (UHR-OCT),^2^ it is possible to resolve additional outer retinal bands^3^ that are not visible with standard-resolution commercial OCT systems. Using UHR-OCT, it is now possible to measure time-dependent changes of photoreceptor-specific structures after light exposure, and then to measure subsequent recovery of the structural changes as an alternative to classical dark adaptometry.

Dark adaptation, in general, is the delayed recovery of light sensitivity in darkness following prior light exposure. Early studies demonstrated that light sensitivity recovers more slowly with increasing bleaching exposures.^4,5^ After sufficiently strong bleaching, two distinct regimens of sensitivity recovery are observed during dark adaptation. Initially, there is a rapid recovery associated with cone photoreceptors that takes place on the scale of minutes and ends in a plateau. Then, a dark adaptation phase associated with rod photoreceptors begins at ∼15 minutes after light exposure in normal subjects and, depending on the bleach level, can continue for up to an hour. Delayed dark adaptation is well established as symptom of a variety of ocular diseases^6,7^ including age-related macular degeneration (AMD), a leading cause of blindness in developed countries. Prior work has shown detectable changes in dark adaptation using perimetry^8^ and single-spot dark adaptometry^9–11^ in AMD patients. Recently, a commercial dark adaptometer (AdaptDX; MacuLogix, Inc., Middleton, MA, USA) has been used to assess the severity of AMD by measuring the delayed dark adaptation before progression to visual acuity loss.^12,13^ Delayed dark adaption thus may be able to serve as biomarker for early AMD progression in clinical trials.

In this study, we used UHR-OCT to investigate changes in the length of photoreceptor outer segments in normal subjects in response to a bleaching exposure, and the subsequent recovery from these changes as a potential surrogate for dark adaptation. Recently, full-field OCT has been used to examine the human cone length changes immediately after patterned flash exposure.^14^ A previous study had investigated the photoreceptor changes when transitioning from darkness to constant light adaptation in normal subjects and Best disease patients using a commercial OCT device.^15^ Other studies have used UHR-OCT to investigate outer retinal band changes of frogs^16^ and mice^17^ between constant light- and the dark-adapted states and of dark-adapted mice undergoing flash stimulus.^18^ Our UHR-OCT study examined the in vivo thickness changes of human outer retinal bands during 30 minutes of dark adaptation after bleaching flashes of varied strength. A critically important feature of the near-infrared light employed for most OCT systems is that it negligibly activates visual pigment. Thus, with the 870-nm-wavelength UHR-OCT system, it is possible to probe the outer retina without altering the physiological state of rods and cones. We discovered three distinct time-dependent thickness changes (photoresponses) whose magnitude and duration were similarly dependent on the bleach level. The outer retinal layer thickness changes of cone and rod photoreceptor bands had distinct time courses that were similar to that of cone- and rod-mediated recovery of sensitivity measured by classic dark adaptometry.

## Methods

### Bleaching Flash Illuminator

Flash bleaching was performed with a high-power 528-nm-wavelength LED (LZ4-00G108; LED Engin, San Jose, CA, USA) diffusely illuminating the retina through a 4F Maxwellian view illuminator.^19^ The illuminator was built with two 60-mm focal length condenser lenses to uniformly expose a 40° retinal field. For patient fixation, a black 1-mm-thick crosshair in the focal plane between the lenses produced a 0.5° thick crosshair on the retina. For the half-field exposure, illumination was restricted to the inferior retina by placing a black cardstock semicircle in the focal plane. The LED exposure intensity and duration was controlled by a precision current source (B2902A; Keysight Technologies, Santa Rosa, CA, USA). The exposure levels were measured with a photometer (ILT1700; International Light Technologies, Peabody, MA, USA) with the SED033/Y/W and SED033/ZCIE/W detectors for photopic and scotopic measurements, respectively. Since the image of the LED source at the iris is smaller than the dilated pupil, all of the light enters the eye. From measurements of the photopic energy (lux-seconds) of the flash exposure at a known distance and the flash durations, we determined the temporally integrated retinal illuminances of the bleaching stimuli in Troland-seconds (Td⋅s)^20^.

**Table:**
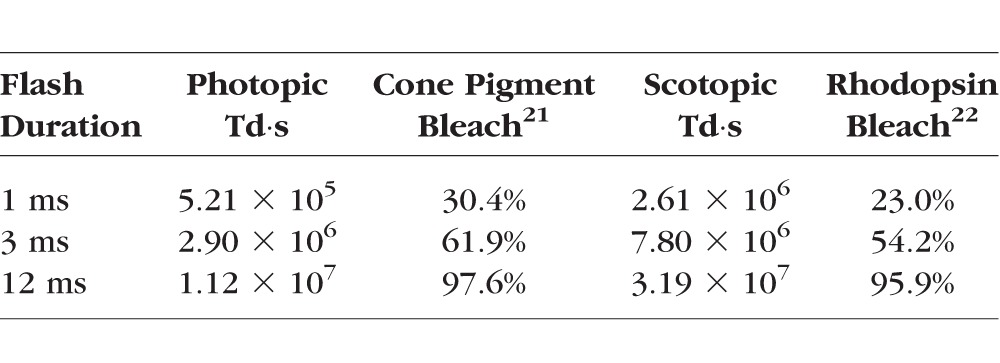
Bleaching Flash Parameters

### Ultrahigh-Resolution OCT System

The prototype ultrahigh-resolution spectral-domain OCT (UHR-OCT) system was developed at the Massachusetts Institute of Technology (MIT) and used a broadband superluminescent diode laser (T870-HP; Superlum Diodes Ltd, Carrigtwohill, Ireland) centered at 870-nm wavelength with full-width half-maximum (FWHM) bandwidth of 170 nm, resulting in a FWHM axial resolution of 4 μm in air and 3 μm in tissue. An 80/20 optical fiber coupler divided the power into the sample and reference arms. Galvanometer scanning mirrors performed retinal scanning, and the 1/e^2^ beam diameter on the cornea was 1.8 mm. The measured power at the cornea was 840 μW, below the American National Standard Institute (ANSI) laser retinal exposure limit.^23^ A dichroic mirror was used in the sample arm to introduce a 640-nm-wavelength LED fixation target positioned at the center of the OCT scan. The UHR-OCT system used a custom spectrometer with an 82-mm focal length collimator incident onto a 1200 line/mm transmission grating, followed by a custom 100-mm focal length lens that focused the diffracted wavelengths onto a line scan camera (spL4096-140km; Basler AG, Ahrensburg, Germany). The camera operated with 3072 pixels for a 91,000 A-scan per second imaging speed and had imaging ranges of 2.86 mm in air and 2.14 mm in tissue. The measured system sensitivity was 96 dB.

### Dark Adaptometer

The dark adaptometer was integrated into the OCT sample arm. A flat LED at 505-nm wavelength illuminated a square 2.8° × 2.8° field centered at 13.5° inferior to the fovea in the same focal plane as the fixation target, similar to previous work by Pugh.^5^ The LED brightness was current controlled by the precision current source used for flash exposure. The subject adjusted the LED current over 6 log orders. A measured calibration curve was used to convert the LED current to stimulus light intensity. During the measurements, the stimulus LED was pulsed at 1 Hz with a 10% duty cycle.

### Subjects and Imaging Protocol

Five normal subjects of ages 26, 28, 28, 33, and 35 years were recruited for the study. All subjects had less than ±6 diopters correction and underwent a standard ophthalmic eye exam to verify that they had no ocular pathologies. Signed informed consent was obtained from all subjects prior to the study. The study protocol was approved by the MIT Committee On the Use of Humans as Experimental Subjects (COUHES) and followed the tenets of the Declaration of Helsinki.

Prior to imaging, the left eye was dilated with 1% tropicamide and covered with a rigid, opaque eye patch for 40 minutes. Imaging was performed in a windowless room, and stray light from electronic equipment was masked. The imaging system operator's monitor was shrouded with blackout fabric. All imaging was performed between 10 AM and 5 PM. After the subject was seated, the room lights were turned off and the eye patch was removed. To verify dilation before the flash exposure, the pupil size was measured with a camera (Lifecam Studio; Microsoft, Seattle, WA, USA) imaging telecentrically through a 30-mm lens focused on the iris and pupil and illuminated by a pair of 750-nm-wavelength LEDs.

Subjects were imaged with the UHR-OCT system using a 6 × 6-mm, 400 × 400 A-scan volumetric scan acquired in 2.1 seconds per volume. Prior to flash exposure, three baseline volumes were obtained in darkness. After flash exposure, OCT volumes were acquired rapidly at approximately 20-second intervals over 5 minutes to observe the initial response. After the initial 5 minutes, the volumes were acquired every minute up to 30 minutes to investigate the long-term response. Consecutive imaging sessions for a subject on a given day were separated with a dark adaptation of at least 40 minutes to minimize the effects from the previous exposure.

The dark adaptometry measurements used the same dilation and initial dark adaptation procedure, but were acquired in separate sessions from the OCT imaging. The subject determined the light sensitivity threshold by fixating on the center fixation target and adjusting the LED current until the offset stimulus LED was not visible. After each measurement, the brightness was increased from the recorded value before repeating the procedure. This measurement was also performed prior to flash exposure in order to determine the dark-adapted threshold. After the flash exposure was applied, the subject performed the threshold light sensitivity measurements at roughly 1-minute intervals up to 30 minutes after the flash exposure.

### OCT Image Processing

Recorded OCT spectra data were resampled to a uniform wavenumber space, and numerical dispersion compensation^24^ was applied to correct for dispersion variations with eye lengths. The data were then zero-padded to 4096 axial pixels per OCT image, corresponding to an axial sampling of 0.52 μm per pixel.

**Figure 1:**
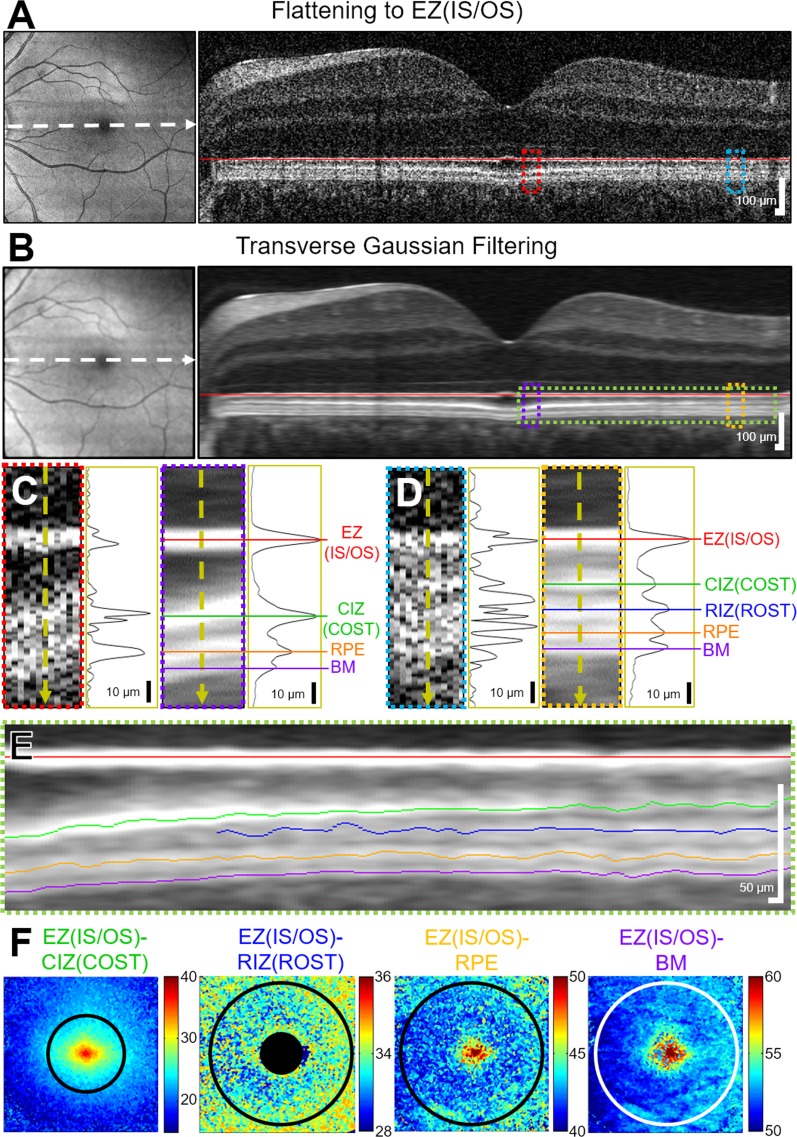
Imaging processing and peak detection steps for volumes acquired at each time point. En face projection and log-scale single B-scan flattened to the ellipsoid zone (EZ), also known as the inner segment/outer segment (IS/OS) junction, from (A) a flattened 6 × 6-mm, 400 × 400 A-scan volume before and (B) after 2D transverse Gaussian filtering at every axial depth in the volume. Indicated portion of the B-scan and a single linear-scale A-scan (C) near and (D) far from the foveal pit demonstrate the increased ability to resolve the backscattering bands after transverse filtering. The resolvable outer retinal bands are the cone interdigitation zone (CIZ), also known as the cone outer segments (COST); rod interdigitation zone (RIZ), also known as the rod outer segment tips (ROST); apical retinal pigment epithelium (RPE); and the Bruch's membrane (BM). (E) Colored outer retinal bands identified by intensity peak detection overlaid on the B-scans. (F) Application of the peak detection to all B-scans produced a thickness map for the four bands relative to the EZ(IS/OS). Numbers associated with the color bars give depths in micrometers relative to the EZ(IS/OS).

As there are differing interpretations of the outer retinal bands seen with OCT,^25–27^ the bands will be described using both nomenclature conventions. The logarithmic-scale OCT intensity image was analyzed with a two-dimensional (2D) graph-cut algorithm based on a previous implementation^28^ to segment the inner segment ellipsoid zone (EZ), also referred to as the inner segment/outer segment (IS/OS) junction, on a B-scan basis. The A-scans within each B-scan were aligned to the EZ(IS/OS) segmentation to produce a flattened EZ(IS/OS) contour. Sequential B-scans were aligned to the EZ(IS/OS) to produce an axially aligned volume (Fig. 1A). A 2D transverse Gaussian filter with a standard deviation of 2 A-scans was applied to every axial depth (C-scan) after flattening to generate A-scans with reduced noise and accentuate the four outer retinal bands below the EZ(IS/OS) (Figs. 1B–D). The first band below the EZ(IS/OS) was identified as the cone interdigitation zone (CIZ), also known as the cone outer segment tips (COST). UHR-OCT resolved the next three distinct bands that often appear as single band in standard-resolution OCT systems. Below the CIZ(COST), the rod interdigitation zone (RIZ), also known as the rod outer segment tips (ROST), starts appearing in the perifovea. The other two layers are the apical retinal pigment epithelium (RPE), and the Bruch's membrane (BM). A peak detection algorithm was used to determine the position of these intensity peaks relative to the EZ(IS/OS) for each filtered A-scan. The interpretation of these bands was determined based on previous histologic^25^ and adaptive optics (AO) OCT literature.^29^

The thickness of the four bands relative to the EZ(IS/OS) for all B-scans comprising each volume were used to generate thickness maps for each time point (Fig. 1F). To determine the photoresponses, each postbleach thickness map was compared by differencing the average thickness maps derived from three volumes taken at the baseline (dark-adapted) condition. Further analysis was performed by averaging the EZ(IS/OS)-CIZ(COST) thickness maps within a 3-mm-diameter circle centered on the fovea in order to include the region with high concentration of cones in the fovea. Similarly, the EZ(IS/OS)-RIZ(ROST) thickness map was averaged within a 1.5- to 5.5-mm-diameter ring to include the parafoveal region with higher concentration of rods. The EZ(IS/OS)-RPE and EZ(IS/OS)-BM thickness maps were averaged within a 5.5-mm-diameter circle. In the patterned illumination study, the areas averaged were further split into inferior and superior halves for comparison of changes in the exposed versus the unexposed regions, respectively.

## Results

### Ultrahigh-Resolution OCT and Dark Adaptometer After Full-Field Bleach

**Figure 2:**
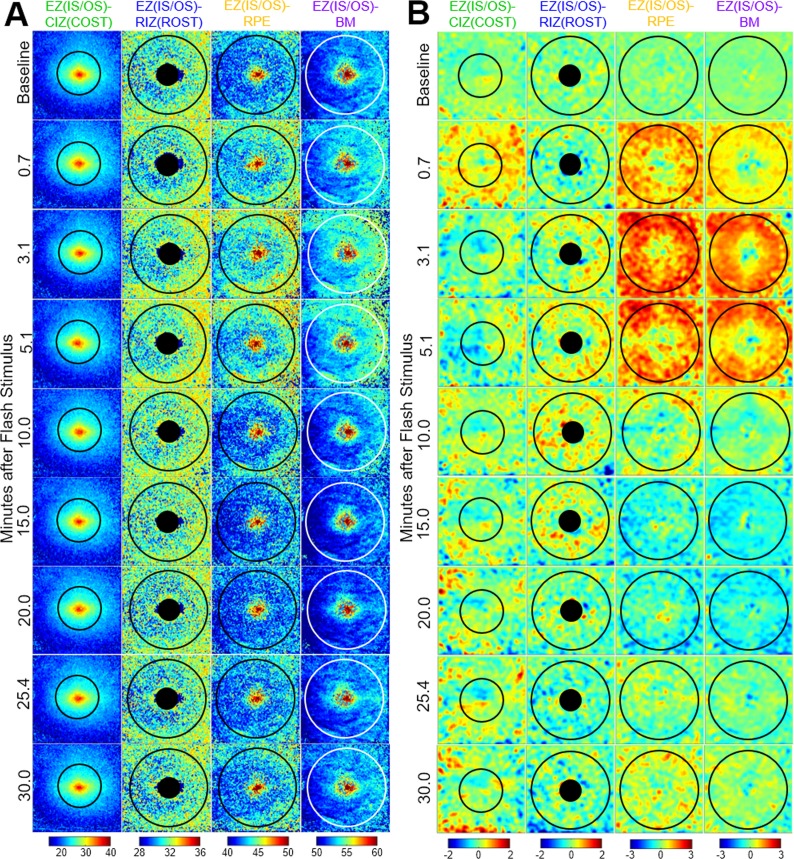
(A) Outer retinal band thickness maps from a single subject at baseline and selected time points after a 96% full-field rhodopsin bleach. Numbers associated with the color bars give depths in micrometers relative to the EZ(IS/OS). (B) Thickness difference maps between each time point and the averaged baseline. A 2D Gaussian filter with standard deviation = 9 A-scans was applied to the thickness difference maps to emphasize regions of change. Numbers associated with the color bars are the difference from baseline in micrometers. The overlaid circles indicate the area used to calculate the average thickness for each band.

The left eyes of the five subjects were imaged with the described UHR-OCT protocol in the dark-adapted condition, and at various times after full-field exposures that bleached 23%, 54%, and 96% of the rhodopsin. Of the 822 UHR-OCT volumes acquired in total from all the subjects during the full-field experiment, two volumes were excluded due to excessive motion artifacts. Recovery of sensitivity measured with the dark adaptometer following full-field rhodopsin bleaches of 23%, 54%, and 96% for one subject is also provided. Figure 2A shows the thickness maps from the EZ(IS/OS) to the four detected bands at selected time points from a single subject during the full-field 96% rhodopsin bleach exposure. Figure 2B shows the thickness difference maps of the same time points after aligning the maps to the fovea, subtracting the averaged baseline thickness map, and applying a 2D Gaussian filter to emphasize areas of change.

**Figure 3:**
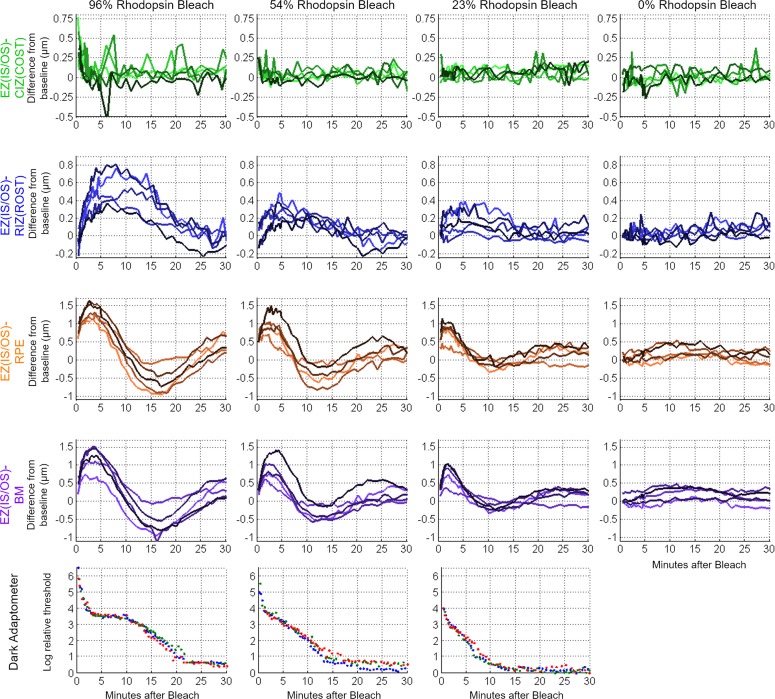
UHR-OCT averaged thickness map area differences (photoresponses) from baseline of the four outer retinal bands relative to the EZ(IS/OS) in five subjects for full-field 96%, 54%, 23%, and 0% rhodopsin bleaches. At the bottom, the results from three sessions using the dark adaptometer on one subject with the same bleach protocol at 96%, 54%, and 23% rhodopsin bleaches are plotted to compare against the UHR-OCT results.

The color-coded difference maps reveal several distinctive changes in layer thicknesses, including parafoveal increases in the EZ(IS/OS)-RPE and EZ(IS/OS)-BM in the initial 5 minutes after the bleach, followed by decreases in thickness at 15 to 20 minutes. A similar, but much more rapid biphasic change in thickness of the EZ(IS/OS)-CIZ(COST) layer is also clearly visible. To further analyze these data, and those from other bleaching exposures, we plotted space-averaged data as a function of time to analyze the photoresponses of all five subjects (Fig. 3).

**Figure 4:**
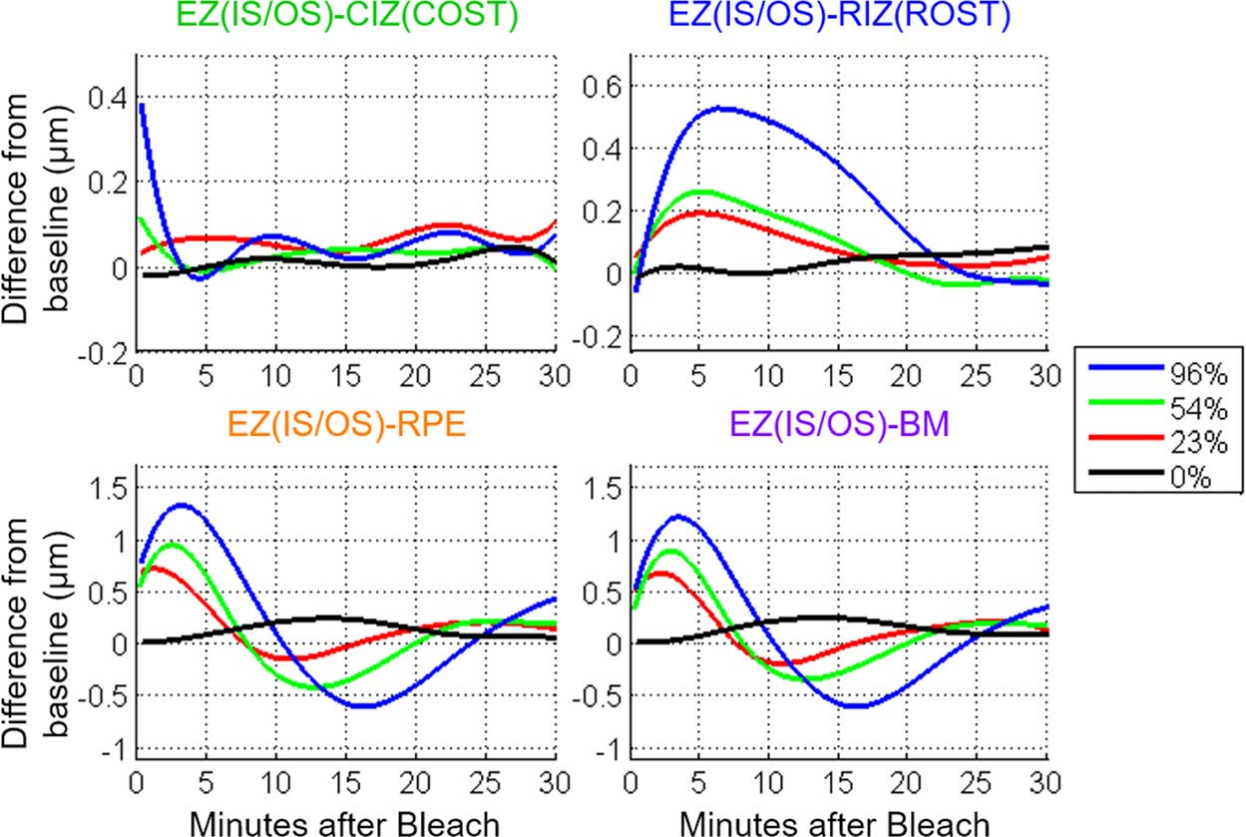
Spline-fitted curves of the photoresponses from the five subjects used to compare the bleach responses (96%, 54%, and 23% rhodopsin bleach) to the control (0% rhodopsin bleach) response.

The results obtained by spatial averaging strongly confirm and extend the conclusions drawn from the thickness difference maps. Using the GLIMMIX procedure in SAS (SAS Institute, Inc., Cary, NC, USA) for statistical analysis, the 96%, 54%, and 23% rhodopsin bleach results were compared against the 0% control data as reference using spline fitting (Fig. 4). The EZ(IS/OS)-CIZ(COST) response curves were significant for the 96% and 54% bleach photoresponse curves (*P* < 0.0001 and *P* = 0.006, respectively). The EZ(IS/OS)-CIZ(COST) 23% bleach response curve was not significant (*P* = 0.43). For the EZ(IS/OS)-RIZ(ROST), -RPE, and -BM responses, all three bleach levels had significant differences in the curves as compared to the control (*P* < 0.0001). The major increases in the EZ(IS/OS)-CIZ(COST) 96% and 54% bleach data occurred at the earliest times after the bleach, followed by a rapid decline to baseline that was completed in less than 5 minutes. In contrast, the EZ(IS/OS)-RIZ(ROST) photoresponses required 5 to 7 minutes to reach their maxima, and then recovered to baseline over times ranging from 15 minutes (23% bleach) to 30 minutes (96% bleach). The EZ(IS/OS)-RPE and EZ(IS/OS)-BM data exhibit similar biphasic responses that are qualitatively distinct from either the EZ(IS/OS)-CIZ(COST) or EZ(IS/OS)-RIZ(ROST) data, reaching peak thickness increases approximately 3 minutes after the bleach and undershooting the baseline at 10 to 15 minutes depending on the bleach level. The dark adaptometry results measured in the same apparatus exhibit the classic features of a cone component that is completed in less than 3 minutes and a much slower rod component that required 15 to 20 minutes to reach completion, depending on the bleach level.

**Figure 5:**
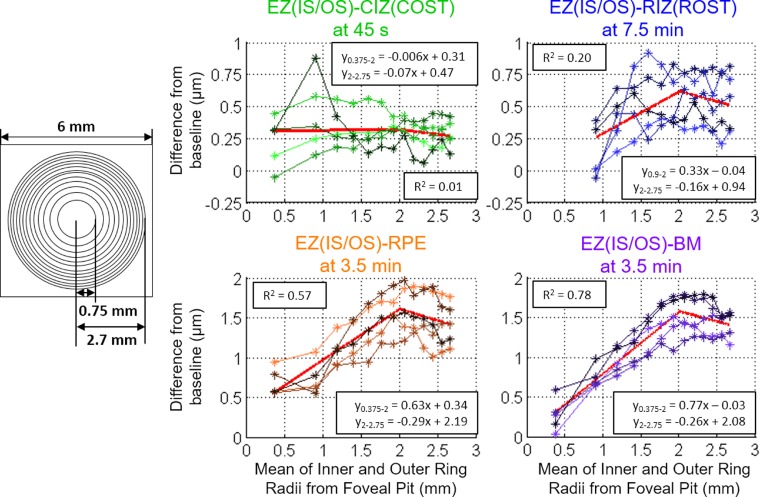
Analysis of the radial changes by averaging the thickness difference of (left) concentric, nonoverlapping rings of equal area at the maximum thickness increase time point for 96% rhodopsin bleach. Right: The results from each of the five subjects are plotted as connected points on the scatter plots. The plots include piecewise linear lines with a knot at 2 mm generated from the mixed-effects analysis.

The spatial averaging employed in generating the results in Figure 3 could mask variations in thickness differences that may be a function of the distance from the foveal pit. To investigate possible radial variations, the average thicknesses were compared to baseline in concentric nonoverlapping rings having an area equal to that of a 0.75-mm-radius circle centered at the foveal pit (Fig. 5). This analysis was performed for the results at the listed time point of the maximal thickness increase for the four bands. Because the responses have two distinct regions, a mixed-effects piecewise linear regression model with a knot (flexible bend) at 2 mm was applied to the results to analyze the thickness change relationship to radius. The linear slope coefficient was statistically significant for 0.75 to 2 mm in the EZ(IS/OS)-RIZ, -RPE, and -BM (*P* < 0.0001). In the second 2- to 2.75-mm segment, the linear slope coefficient was significant in the EZ(IS/OS)-RPE and -BM (*P* = 0.002 and *P* = 0.009 respectively) but not in the EZ(IS/OS)-RIZ(ROST) (*P* = 0.069). The EZ(IS/OS)-CIZ(COST) linear slope coefficient was not significant for both segments (*P*_0.75-2_ = 0.87, *P*_2-2.75_ = 0.36). We emphasize that the purpose of this analysis was to examine the data for radial trends, and was not intended to represent a mechanistic model.

### Ultrahigh-Resolution OCT Half-Field Bleach

**Figure 6:**
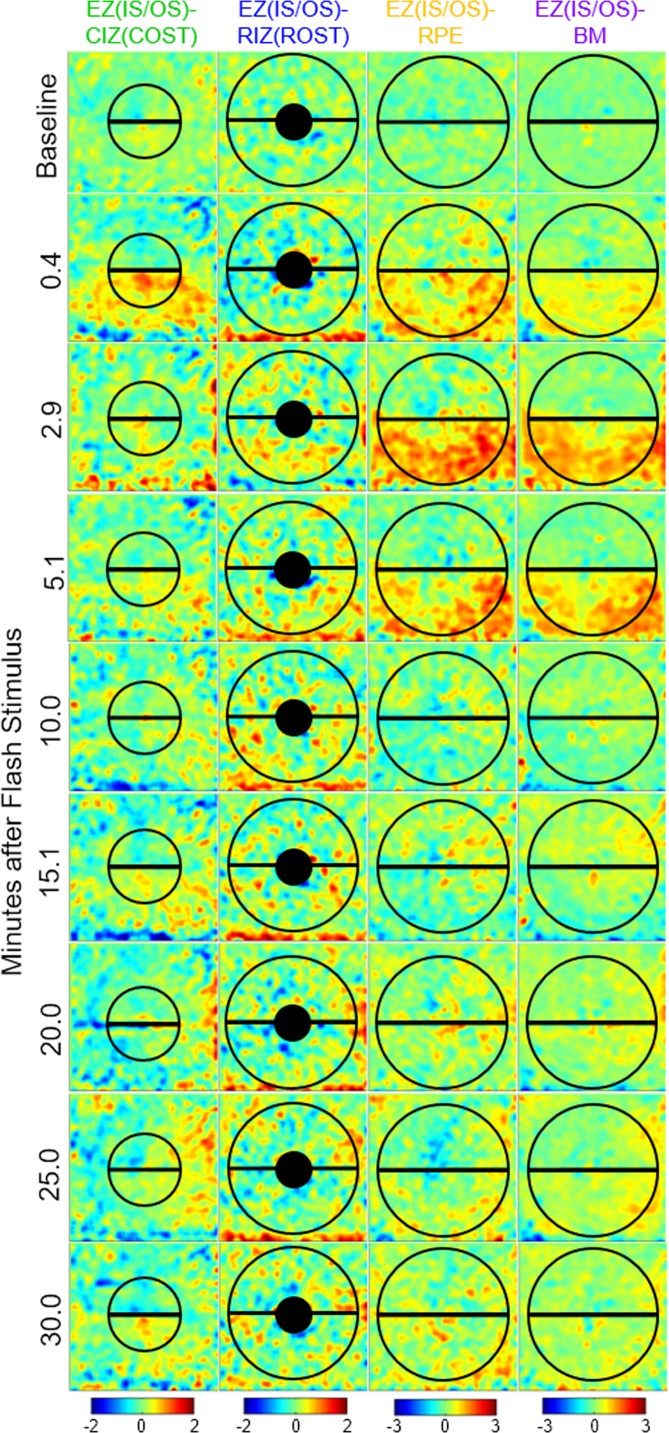
Thickness difference maps from a single subject at selected time points after the 54% half-field rhodopsin bleach. A 2D Gaussian filter with standard deviation = 9 A-scans was applied to the thickness difference maps to emphasize regions of change. The semicircle areas indicate the area for the unexposed (superior retina) and exposed (inferior retina) regions used to calculate the average thickness for each band at every time point. Numbers associated with the color bars are the micron difference from baseline.

**Figure 7:**
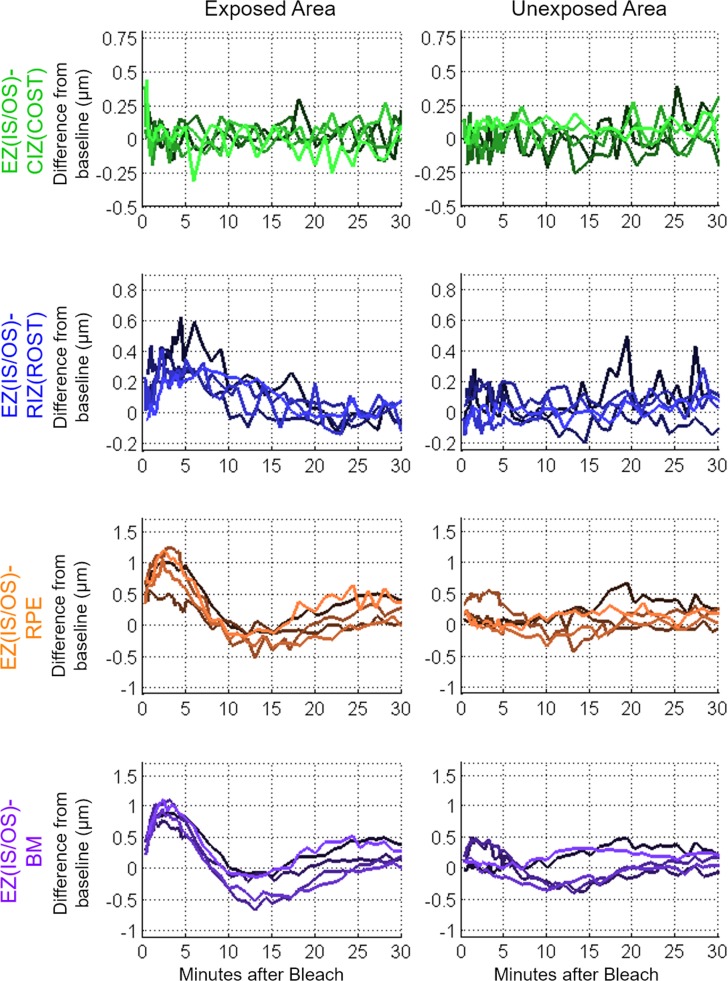
Averaged thickness map area differences (photoresponses) from baseline in the five subjects for half-field 54% rhodopsin bleach. The exposed (inferior retina) and unexposed (superior retina) half-fields are plotted separately.

The left eyes of the five subjects were imaged with the half-field bleach at 54% rhodopsin bleach exposure to determine if the thickness changes were localized. Of the 210 UHR-OCT volumes acquired in total from the half-field bleach sessions, no volumes were excluded for motion artifacts. Figure 6 shows a single subject's thickness difference map at selected time points with areas of averaging indicated. The photoresponses of the exposed and unexposed areas are plotted over time in Figure 7.

**Figure 8:**
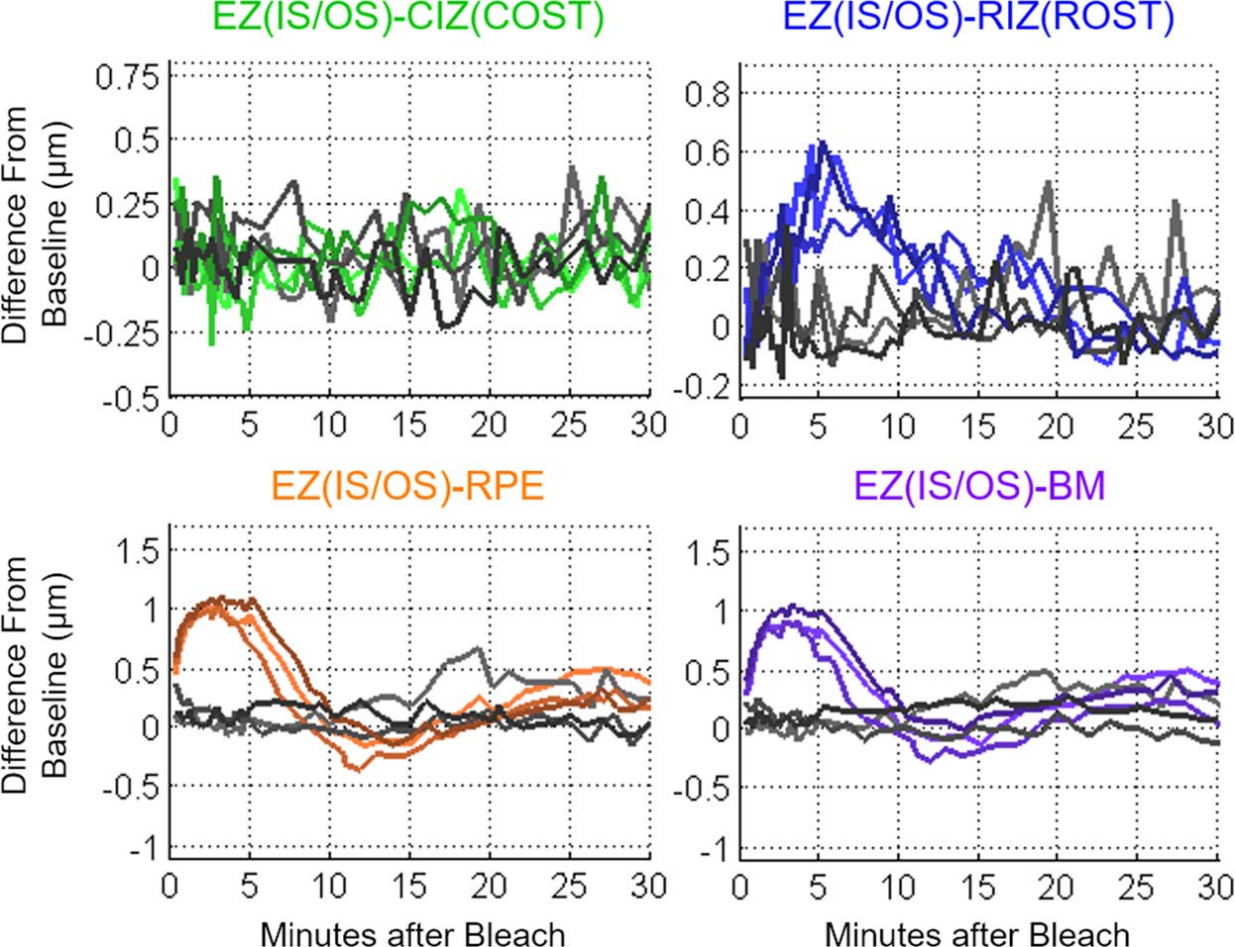
Averaged thickness map area differences (photoresponses) from baseline of the half-field 54% rhodopsin bleach exposure repeated three times for a single subject. Colored curves indicate the data from the exposed half-field, and the grayscale curves indicate the data from the unexposed half-field.

The GLIMMIX statistical analysis was performed on the splines fitted to the thickness results from the exposed versus the unexposed hemifield. The exposed half-field EZ(IS/OS)-CIZ(COST) thickness changes statistically differed from that in the unexposed half-field (*P* = 0.006). Additionally, the exposed half-field EZ(IS/OS)-RIZ(ROST), -RPE, and -BM thickness changes all statistically differed from that of the unexposed half-field (*P* < 0.0001). One subject was imaged an additional two half-field bleach sessions to analyze repeatability among the three sessions. The results of the three sessions are plotted in Figure 8. Overall, these results demonstrate that the layer thickness changes are confined to the retinal region exposed to the bleaching stimulus.

## Discussion

The UHR-OCT imaging results presented in the current study revealed distinct bleach-dependent outer retinal thickness changes of the EZ(IS/OS)-CIZ(COST), -RIZ(ROST), -RPE, and -BM intervals (Fig. 2), and allowed the time courses of recovery or dark adaption of these photoresponses to be measured (Fig. 3). The magnitude and the duration of the responses increased with bleach intensity, and the time courses were comparable to cone and rod components of dark adaptation curves measured under the same conditions. Experiments in which the bleaching stimuli were applied to only half the visual field revealed that the changes were localized to the region bleached, rather than a general change in the retina (Figs. 6, 7). Measurements on multiple subjects and repetitions with a single subject showed that the maximal magnitude layer thickness changes and their recovery time courses were highly replicable across subjects and conditions.

### Properties of Cone Interdigitation Changes Manifest in EZ(IS/OS)-CIZ(COST) Data

The EZ(IS/OS)-CIZ(COST) thickness changes are attributable to changes in the cone outer segments, based on the depth location of the OCT bands and the rapid recovery to baseline within 5 minutes of the 96% and 54% bleaching exposures. The magnitude of these cone-specific responses scaled with percent pigment bleached, such that the maximal length increase after the 96% bleach was approximately twice that following the 54% bleach. Cone spatial density and the lengths of cone outer segments are known to be decreasing functions of the distance from the foveal pit. However, the EZ(IS/OS)-CIZ(COST) thickness increase at the time of its maximal increase appeared independent of the radial distance from the foveal pit (Fig. 5). A factor that possibly contributes to this discrepancy is that radial variation may be obscured by the fixation crosshairs in the bleaching apparatus, which occupy the center of vision where cone density and axial length are greatest. Furthermore, after 1-mm (∼3°) retinal eccentricity, the cone photoreceptor density decreases more slowly than the rapid increase in the rod photoreceptor density,^30^ so cell density changes may be less pronounced. Future studies with higher statistical power from more subjects and more precise imaging with AO-OCT will be necessary to discern if the cone photoresponse varies with radial distance.

Recently, Hillman et al.^14^ used full-field OCT to measure EZ(IS/OS)-CIZ(COST) thickness increases of single cones within 3 seconds of 10-μW radiant flux white light flash exposures in healthy humans. By analyzing the phase changes of the OCT data, they were able to resolve localized submicron thickness changes of the EZ(IS/OS)-CIZ(COST) immediately after patterned illumination. Although the absolute bleaching levels could not be calculated to compare with our study, their work showed similar EZ(IS/OS)-CIZ(COST) thickness increases that scaled with exposure intensity. Given the immediate thickness changes in the Hillman et al. study and the rapid decay of the EZ(IS/OS)-CIZ(COST) response shown in our study, the OCT volume temporal sampling used in our investigation may underestimate the recovery magnitude somewhat. Future studies of the bleaching exposure effects on cones should use a more rapid rate of acquisition immediately after flash.

Previous AO-OCT imaging of cone photoreceptors in humans showed shortening of cone outer segments that was interpreted as RPE cell phagocytosis of the outer segments, that is, disk shedding.^31^ This change is opposite in sign to the increased thickness change observed in the EZ(IS/OS)-CIZ(COST) band, and was not demonstrated to be bleach dependent. Furthermore, based on histologic rodent studies^32–37^ and AO-OCT human experiments,^31^ disk shedding is linked to a circadian cycle and most actively occurs in the early morning near the time of normal light onset. Our imaging study was performed in the late morning to afternoon to avoid peak periods of disk shedding.

### Features of Rod Interdigitation Changes Revealed by the EZ(IS/OS)-RIZ(ROST) Data

The EZ(IS/OS)-RIZ(ROST) thickness changes are attributable to changes in the rod outer segments by a logic parallel to that used to attribute EZ(IS/OS)-CIZ(COST) changes to cone outer segments, namely, by prior band assignments made from histology and by the much slower kinetics (Fig. 3). In addition, the thickness changes scale with a radial distribution that matches the increasing rod photoreceptor density with retinal eccentricity (Fig. 5). (An additional rationale for this assignment of the EZ(IS/OS)-RIZ(ROST) changes comes from recent work in mice, which is discussed below.) A striking and highly distinctive feature of the EZ(IS/OS)-RIZ(ROST) changes is the relatively long time after the bleaching exposure (∼5 minutes) that it takes for these increases to reach their maxima. This time course contrasts sharply with the very brief period (on the time scale of our experiments) required for the EZ(IS/OS)-CIZ(COST) changes to reach their maxima. Also notable is that the slow recovery of the EZ(IS/OS)-RIZ(ROST) changes to their dark-adapted level roughly parallels the time course of recovery of rod sensitivity determined with dark adaptometry.

Some changes in the cone and rod interdigitation zones could arise from light-driven melanosome movement in the RPE. Zhang et al.^16^ in 2013 examined RPE melanosomes in leopard frogs during dark- and light-adapted conditions with OCT and histology. They found that the melanosomes migrated to the apical projections of the RPE in light-adapted frogs, causing a statistically reliable increase of the hyperreflective photoreceptor bands compared with dark-adapted frogs. However, if light exposure were to drive human melanosomes toward the apical RPE as observed in frogs, there would be a net decrease in the EZ(IS/OS)-RIZ(ROST) thickness, opposite to the direction of change observed in our experiments.

### Rod Outer Segment Changes Observed in the EZ(IS/OS)-RPE and EZ(IS/OS)-BM Data

The EZ(IS/OS)-RPE and EZ(IS/OS)-BM responses were nearly identical, suggesting that the thickness of the RPE cells themselves is negligibly changed by the bleaching exposures. This in turn further strengthens the conclusion that the underlying mechanism of the EZ(IS/OS)-RIZ(ROST) thickness changes resides anterior to the RPE in the rod outer segments themselves. Similar to the EZ(IS/OS)-RIZ(ROST) response, the EZ(IS/OS)-RPE/BM responses increased radially, suggesting that the thickness changes may also be due to increasing rod cell densities. At all bleach levels used, the EZ(IS/OS)-RPE/BM responses were statistically reliable. Despite the similarities of the radial profiles of their maxima (Fig. 5), the time courses of the EZ(IS/OS)-RPE/BM responses differed from that of the EZ(IS/OS)-RIZ(ROST) response (Figs. 3, 4). The two responses had an initial increase in thickness which quickly peaked. Both the peak magnitude and time to reach the peak scaled with bleach intensity. Following the peak, the thickness decreased to below the baseline for the higher bleach levels. After reaching a trough, the response oscillates back to a positive thickness change. The dampened oscillation eventually converges to the baseline value for the lower bleach intensities. This response waveform has been observed in direct current (DC) electroretinogram (ERG) and electrooculogram (EOG) literature where a voltage peak (light peak) occurs 5 to 10 minutes after light exposure, followed by a dampened oscillation associated with ion gradient changes in the RPE.^38–40^ The light peak-to-trough amplitude scales with the log light intensity,^41^ matching how the EZ(IS/OS)-RPE/BM photoresponses scaled with bleach intensity. Abramoff et al.^15^ compared EOG results with photoreceptor outer segment thickness changes observed with a commercial OCT system (Heidelberg Spectralis; Heidelberg Engineering, Heidelberg, Germany) in normal humans and patients with Best vitelliform macular dystrophy during dark to light adaptation. Their study found significant shortening of the outer segments during light adaptation in normal subjects that matched the EOG response. Although their study used constant light instead of a single flash, the shortening of the outer segments at ∼15 minutes after light adaptation is consistent with the thickness reduction below baseline in the EZ(IS/OS)-RPE/BM observed in our study. The limited 7 μm axial resolution of the Heidelberg Spectralis may blur the RIZ(ROST), RPE, and BM bands together as a single band such that only the largest changes were observed. Furthermore, the imaging interval of 4 minutes between OCT data sets in their study may not have had the temporal resolution to capture the light peak and instead, only observed the trough of the oscillating response.

### Potential Mechanism of Outer Segment Elongation: Osmotic Swelling

On the hypothesis that the thickness changes observed by UHR-OCT primarily manifest as physical changes in the length of outer segments, the issue of the cellular mechanism of bleach-dependent outer segment elongation comes to the fore. Two recent OCT studies of mice are pertinent to this issue. Li et al.^17^ in 2016 reported a light-stimulated increase in the EZ(IS/OS)-RIZ(ROST) distance in pigmented (C57Bl/6) mice observed with a 1.6-μm axial-resolution UHR-OCT system (Envisu UHR2200; Bioptigen, Durham, NC, USA), and directly confirmed with histology that the length of rod outer segments (ROS) increased correspondingly. Zhang et al.^18^ in 2017 investigated both pigmented (C57Bl/6) and unpigmented (Balb/c) mice with UHR-OCT (∼2-μm axial resolution), and showed that the two strains exhibited nearly identical bleach-dependent increases in the length of the ROS. (Due to the absence of melanosomes, the RPE–rod outer segment interdigitation zone is not detectable with OCT in albino mice.) The time course of increase of rod outer segment length in the latter study was remarkably similar to that observed in this study of the human EZ(IS/OS)-RIZ(ROST) and EZ(IS/OS)-RPE/BM: After exposures that bleached 10% to 100% of the rhodopsin it took several minutes for ROS elongation to reach its maximum. However, the study observed the photoresponse for only 5 minutes after flash bleaching, so the recovery to normal length was not observed for bleaches exceeding 10%. The latter study also showed that in mice lacking the essential rod phototransduction protein transducin (*Gnat1*^−/−^), ROS elongation was completely absent, incontrovertibly establishing that the OCT-measured ROS layer elongation is driven by light captured by rods.

Zhang et al.^18^ in 2017 hypothesized that bleach-dependent ROS elongation is an osmotic swelling response to an increase in outer segment osmolarity triggered by phototransduction and established that the slow time course of swelling and the inferred water permeability of the outer segment membrane were consistent with the hypothesis. Differences between onset and recovery of the swelling time courses in mouse and human rods, and in their bleach-level dependence, suggest important differences in the way mouse and human rods respond to osmotic stress, and are topics of ongoing investigation.

Li et al.^17^ in 2016 shared a similar hypothesis by referencing previous studies of subretinal space (SRS) osmotic changes. This SRS lies between the inner segments/Müller cells and the apical membrane of the RPE. Studies using injected impermeable ions have shown an ion concentration reduction following light stimulation associated with an increased SRS volume due to water diffusion.^42–44^ This light-induced diffusion has also been confirmed using magnetic resonance imaging in rodent eyes.^45,46^ Since ERG and EOG measure the oscillating electric potential changes caused by ion movement of the RPE,^38,40^ the similarities of the EZ(IS/OS)-RPE/BM photoresponses to the ERG and EOG light response suggest that OCT measures the physical variations in hydration associated with ionic potential changes.

### Limitations and Caveats

Although averaging A-scans over an area of the retina enabled detection of submicron changes in the outer segment structures, fluctuations in the measurements limited the ability to detect some of these responses. Some variation may arise from cell-to-cell length differences, from speckle noise in the OCT, or from limitations in the segmentation algorithm. The current study segmented and flattened to the EZ(IS/OS), which may have more variation compared to flattening to the external limiting membrane (ELM) or to BM. However, the EZ(IS/OS) band is more prominent than the ELM in human OCT images, and segmenting to the BM may result in errors because of its proximity to the RPE band. Furthermore, because all the thicknesses were referenced to the EZ(IS/OS), the lack of thickness changes common to all four measurements suggested that the EZ(IS/OS) position did not vary independently. At the lowest bleaching intensity, some of the submicron responses may have been too close to the noise limit for this measurement method. Increasing the transverse resolution through AO-OCT with ultrahigh axial resolution^29^ will reduce the transverse speckle noise and allow for imaging of the response of single photoreceptors. Improvements to the segmentation will allow more precise detection of these localized responses to reduce the need to average large areas. Detecting focal alterations would be useful to assess the areas within and surrounding retinal pathology.

Another limitation of the study is that the sample population was exclusively normal young adults. The responses may be different with a different age group, as dark adaptation recovery is known to become slower with age.^47^ Furthermore, changes in the appearance of the outer segments in pathologic retinas^48–50^ may affect the ability to resolve these different bands in patients. Future studies to determine whether these photoresponses can serve as markers of rod and cone health will need to measure the responses in patients with diseases such as AMD that delay dark adaptation and in age-matched normal controls.

## Conclusions

We have measured reliable, localized submicron changes in the human photoreceptor bands after flash exposure and subsequent dark adaptation with UHR-OCT. These changes vary with bleaching level in a manner similar to sensitivity recovery measured with classic dark adaptometry. The findings confirm prior reports in humans and animals of outer segment lengthening following strong light exposure, and recovery during subsequent dark adaptation. These dynamic changes warrant further investigation as possible markers of photoreceptor health.
